# Comparison of enzymatic activities and proteomic profiles of *Butyrivibrio fibrisolvens* grown on different carbon sources

**DOI:** 10.1186/s12953-019-0150-3

**Published:** 2019-06-01

**Authors:** Hana Sechovcová, Lucie Kulhavá, Kateřina Fliegerová, Mária Trundová, Daniel Morais, Jakub Mrázek, Jan Kopečný

**Affiliations:** 1 Institute of Animal Physiology and Genetics, CAS, v.v.i., Vídeňská 1083, 142 20 Prague, Czech Republic; 2 Institute of Physiology, CAS, v.v.i., Vídeňská 1083, 142 20 Prague, Czech Republic; 3 Institute of Biotechnology, CAS, v.v.i., Průmyslová 595, 252 50 Vestec, Czech Republic; 4 Department of Analytical Chemistry, Faculty of Science, Charles University in Prague, Hlavova 8, 12843 Prague 2, Czech Republic; 5 Department of Biochemistry and Microbiology, Faculty of Food and Biochemical Technology, University of Chemistry and Technology, Technická 5, 166 286 Prague, Czech Republic; 6 Institute of Microbiology, CAS, v.v.i., Vídeňská 1083, 142 20 Prague, Czech Republic

**Keywords:** *Butyrivibrio fibrisolvens*, Proteomics, Rumen, Carbon sources

## Abstract

**Background:**

The rumen microbiota is one of the most complex consortia of anaerobes, involving archaea, bacteria, protozoa, fungi and phages. They are very effective at utilizing plant polysaccharides, especially cellulose and hemicelluloses. The most important hemicellulose decomposers are clustered with the genus *Butyrivibrio*. As the related species differ in their range of hydrolytic activities and substrate preferences, *Butyrivibrio fibrisolvens* was selected as one of the most effective isolates and thus suitable for proteomic studies on substrate comparisons in the extracellular fraction. The *B. fibrisolvens* genome is the biggest in the butyrivibria cluster and is focused on “environmental information processing” and “carbohydrate metabolism”.

**Methods:**

The study of the effect of carbon source on *B. fibrisolvens* 3071 was based on cultures grown on four substrates: xylose, glucose, xylan, xylan with 25% glucose. The enzymatic activities were studied by spectrophotometric and zymogram methods. Proteomic study was based on genomics, 2D electrophoresis and nLC/MS (Bruker Daltonics) analysis.

**Results:**

Extracellular β-endoxylanase as well as xylan β-xylosidase activities were induced with xylan. The presence of the xylan polymer induced hemicellulolytic enzymes and increased the protein fraction in the interval from 40 to 80 kDa. 2D electrophoresis with nLC/MS analysis of extracellular *B. fibrisolvens* 3071 proteins found 14 diverse proteins with significantly different expression on the tested substrates.

**Conclusion:**

The comparison of four carbon sources resulted in the main significant changes in *B. fibrisolvens* proteome occurring outside the fibrolytic cluster of proteins. The affected proteins mainly belonged to the glycolysis and protein synthesis cluster.

**Electronic supplementary material:**

The online version of this article (10.1186/s12953-019-0150-3) contains supplementary material, which is available to authorized users.

## Introduction

*B. fibrisolvens* was isolated from rumen fluid in the mid-fifties by Bryant and Small [1]. It is a strictly anaerobic, non-spore-forming, monoflagellated, butyrate producing bacterium, which is a member of the family Lachnospiraceae (order Clostridiales, class Clostridia, phylum Firmicutes). This genus belongs to the core or heritable rumen species, which represent nearly half of the rumen population [2]. Together with the genera *Ruminococcus*, *Butyrivibrio*, and the Christensenellaceae family, all members of Firmicutes, they form an enriched rumen microbiota population in the prepartal period [3]. *Butyrivibrio* together with *Pseudobutyrivibrio* species also belong to the very first 25% colonizers of feed fibre [4]. The total numbers of both groups can amount to 0.6–2% of total bacterial counts [5]. In the colon, their levels fall to 0.001% [6]. Most strains can ferment various soluble sugars, disaccharides and oligosaccharides, producing butyrate as the major end product [7]. This feature makes *Butyrivibrio* strains an important component of digestive tract microbiota influencing the healthy state of colonocytes for which butyrate is the main energy source [8].

In the rumen, butyrivibria represent an important fibre degrader group which are able to utilize various hemicelluloses and xylans, pectin and starch, however exhibiting poor or no growth on cellulose [9]. In grasses, the predominant component of hemicellulose is arabinoxylan, containing β (1 → 4) linked xylose units, with α (1 → 2) and α (1 → 3) linked arabinose units with D-galactose, possibly D-glucose, as well as side chains of L-rhamnose [10]. The enzymes and genes involved in the decomposition of xylan by *Butyrivibrio fibrisolvens* strains have been thoroughly studied [11] and characterized after xylanase cloning and expression in *E. coli* [12] or after its purification from the culture medium [13]. Valuable data coming from the whole genome sequencing and proteome analysis has helped to supply the respective databases (UniProt, Cazy) with relevant information and create an integrated picture of the enzymatic map of this important bacterium for biomass conversion in ruminant animals.

The crucial enzymes of xylan hydrolysis are three types of endo-β-1,4-xylanases (EC 3.2.1.8) from GH family 10, 11, 30 and two types of 1,4-β-xylosidases (EC 3.2.1.37) from GH family 1 and 43 [14]. Endo-xylanase of the GH 10 family requires two unsubstituted xylopyranose units, GH 11 family needs three xylopyranose units in row, and GH 30 family is a xylanohydrolase (glucuronoarabinoxylan endo-β-1,4-xylanase (EC 3.2.1.136)) acting on glucuronoxylan [15]. 1,4-β-xylosidase of family GH 43 is involved in xylooligosaccharide hydrolysis [16] and 1,4-β-xylosidase of family GH 1 splits off xyloside unit from the non-reducing end of xylan chain [17].

The utilization of hydrolytic products from hemicelluloses usually occurs under carbon catabolic repression (CCR), resulting in a preference for hexose over pentoses in bacteria [18]. The mechanism of simultaneous pentose and hexose utilization was observed in thermophilic G+ anaerobes (TGPA). An isolate of *Thermoanaerobacter* sp. utilized both hexose and pentose simultaneously. It was found that its glycobiome is organized into 13 modules (4–138 genes), and these genes are functionally coherent, presumably based on positive co-expression [19]. In Butyrivibrio species, both CCR and simultaneous metabolism were observed. The type strain of *B. fibrisolvens* 3071 is able to utilize xylose and glucose simultaneously [20]. Therefore these substrates were chosen for this proteomic study.

Nowadays, molecular methods for fibre degradation including genomics and proteomics are preferred to obtain a deep understanding of the digestion process [21]. Among ruminal xylanolytic bacteria, advanced proteomic and mass spectrometric methods for exploring xylan degradation have been used exclusively for *B. proteoclasticus*. Its complete genome sequence [22] together with the studies of its extracellular polysaccharide-degrading proteome, its cytosolic oligosaccharide-degrading proteome [23, 24] and its carbohydrate transporting membrane proteins [25] substantially expanded current knowledge about the hydrolytic capability of the *B. proteoclasticus* type strain B3176^T^.

The hydrolytic system of *B. fibrisolvens* is however still unexplored and far less investigated than that of *B. proteoclasticus*. Therefore in this study, we proposed to construct a theoretical secretome of *B. fibrisolvens* 3071 and to compare proteomic profiles of *B. fibrisolvens* 3071 grown on four substrates, complex polysaccharide (xylan), simple pentose (xylose), simple hexose (glucose) and carbohydrate mixture (xylan+glucose), which resulted in six pair-wise comparisons.

## Material and methods

### Bacterial strain, culture conditions and sample preparation

*Butyrivibrio fibrisolvens* strain DSMZ 3071 (*B. fibrisolvens* 3071) was obtained from the DSMZ culture collection. *B. fibrisolvens* 3071 was cultivated to the late stationary phase under anaerobic conditions at 37 °C in medium M10 [26] without rumen fluid. Various substrates including 4% (w/v) D-xylose (Sigma-Aldrich), 4% (w/v), D-glucose (Sigma-Aldrich), 4% (w/v) beechwood xylan (Fluka), and the combination of 3% (w/v) beechwood xylan (Fluka) with 1% (w/v) D-glucose (Sigma-Aldrich) were used as carbon sources. Bacteria were collected by centrifugation at 10000 g for 20 min at 4 °C. The supernatants containing extracellular enzymes were further processed at 10 °C in a stirred ultrafiltration cell (Millipore) using a Millipore PES membrane with a 10 kDa cut off and stored immediately at − 24 °C. The 6-fold concentrated extracellular enzyme extracts were used for monitoring the proteins secreted in response to the different growth substrates.

### Isolation of DNA and genome sequencing

Genomic DNA was isolated from the *B. fibrisolvens* 3071 strain cultivated on DSMZ medium 330 (http://www.dsmz.de/microorganisms/medium/pdf/DSMZ_Medium330.pdf) under anaerobic conditions [27]. DNeasy UltraClean Microbial kit (Qiagen) was used for nucleic acid isolation with a modified lysis step with prolonged incubation at 70 °C. Genomic DNA concentration was determined using a Qubit 2.0 Fluorometer (Thermo Fisher Scientific) and the quality and integrity of DNA were checked by electrophoresis on 0.8% agarose gel. The genome was sequenced, trimmed and assembled with PacBio technology by GATC Biotech (Germany). The contigs of draft genome were processed with Geneious 9.1.8 software (Biomatters Ltd., New Zealand). The processing of output contigs was performed with KEGG’s internal annotation tools [28] and PROKKA software tool [29].

### Enzyme assay

Spectrophotometric determination of xylanase enzyme activity was performed according to Lever [30]. The assay mixture contained 100 μl of culture supernatant, 100 mmol/l phosphate buffer (pH 6) and 0.5% (w/v) carboxymethyl (CM) xylan as a substrate [31] and was incubated for 60 min at 40 °C. The reaction was stopped by 0.3 M Ba (OH)_2_ and 0.3 M ZnSO_4_, and mixture was centrifuged (6000x g,10 min). The PAHBAH reagent was added (0.9 ml) and the mixture was boiled for 10 min. After cooling the reducing sugars were measured spectrophotometrically at 410 nm (Biomate 5, UK).

The activity of β-xylosidase was determined according to Bidochka [32]. The assay mixture contained 100 μl of culture supernatant, 100 mmol/l phosphate buffer (pH 6) and 0.5% (w/v) p-nitrophenyl-β-D-xylopyranoside (*p*-NPX) (P-LAB) as the substrate and was incubated for 60 min at 40 °C. The reaction was stopped by 0.8 ml 2% (w/v) Na_2_CO_3_ and mixture was centrifuged (6000x g, 10 min). The reducing sugars were measured spectrophotometrically at 410 nm (Biomate 5, UK).

The protein concentration was determined according to Bradford [33]. Bovine serum albumin (BSA) solution (Sigma-Aldrich) was used for calibration. Two hundred μl of working solution (Coomassie Brilliant Blue G-250) (Bio-Rad) and 4 μl sample (or standard) were pipetted into the 96 well microplate. The absorbance was measured at 595 nm (Sunrise, Tecan, Scholler) and the concentration of proteins in the sample was calculated using the linear regression method.

### Sodium dodecyl sulphate-polyacrylamide gel electrophoresis (SDS-PAGE) and zymography

SDS-PAGE of crude extracellular extracts of *B. fibrisolvens* 3071 grown in medium with different substrates as described above was performed according to Laemmli [34] on a slab of 8% polyacrylamide gel in a Mini-Protean Tetra cell system (Bio-Rad) at 110 V for 1.5 h. Protein bands were visualized with Bio-Safe Coomassie R-250 Staining Solution (Bio-Rad) and analysed in a GS-700 Imaging Densitometer (Bio-Rad). Zymograms were prepared according to Flint et al. [35] using 0,1% (w/v) carboxy-methyl (CM) xylan as the substrate [31] on a slab of 8% polyacrylamide gel under the same conditions as for SDS-PAGE. The gel was washed with 1% (v/w) Triton X100 (Fluka) for 30 min and three times with 25 mM phosphate buffer (pH 7,5) to allow the renaturation of enzymes. The gel was then incubated for 20 min at 39 °C in 25 mM phosphate buffer (pH 7,5) and stained in 0.1% (v/w) Congo Red (Sigma-Aldrich) for 30 min. The highlighted spots indicated xylanase activity against a red background after destaining with l M NaCl.

### Two dimensional gel electrophoresis

The comparison of four different carbon sources was based on pair-wise matching with two replicates for each substrate (8 gels) (Additional file 2: Table S2, Figures S1-S12). Protein samples were precipitated for 1 h using 10% trichloroacetic acid (w/v) and pelleted by centrifugation at 4 °C, 7500 x g for 15 min. Pellets were washed with 1 ml of ice-cold acetonitrile, incubated with acetonitrile at 4 °C for 1 h, and centrifuged at 4 °C, 7500 x g for 15 min. The washing step was repeated with acetone and acetonitrile, and the pellets were dried for 1 h at room temperature. Samples were resuspended in lysis buffer (7 M urea, 4 M thiourea, 4% (w/v) CHAPS, 0.6% (w/v) Biolyt and 1% (w/v) DTT) to achieve a final protein concentration of 1 mg/ml. One hundred and forty μl of sample was applied to a 7-cm IPG strip with a linear gradient of pH 4–7 (ReadyStripTM IPG Strips, Bio-Rad). Isoelectric focusing (IEF) was carried out on IPG strips using a Protean IEF cell system (Bio-Rad). Before the second dimension step, focused strips were equilibrated for 10 min in 1 ml of equilibration solution (0,375 mM Tris/HCl, pH 8.8, 6 M urea, 20% (v/v) glycerol, 2% (w/v) SDS, 0,005% BPB (bromophenol blue) and 2% (w/v) DTT (dithiothreitol), and then in the same buffer solution supplemented with 4% (w/v) IA (iodoacetamide) instead of DTT. The strips were then rinsed in Tris-glycine buffer (pH 8.5) and the second dimension was performed in a homogeneous 10% SDS-PAGE gel at 110 V for 1.5 h using a Mini-Protean Tetra cell system (Bio-Rad). The gels were fixed and stained with Bio-Safe Coomassie R-250 Staining Solution (Bio-Rad) and scanned using a GS-800™ Calibrated Imaging Densitometer (Bio-Rad). The protein spots were detected and their intensities quantified using PDQuest TM software (version 8.0.1. Bio-Rad). For statistical analysis, the two-tailed Student t-test (*p* < 0.05) was used to assess the statistical significance of changes in protein abundance.

### Mass spectrometry and bioinformatics

Spots indicating significant differences between the used substrates (p < 0.05) were excised from the gels and processed according to Schevchenko et al. [36]. The excised spots were digested in solution containing NH_4_HCO_3_ (0.05 mol/l) and trypsin (0.02 mg/ml) at 37 °C for 16 h. Extracted peptides were purified using Stage Tips [37]. Extracted solutions were lyophilized and dissolved in 20 μl of 2% formic acid (v/v) for consequent nLC MS/MS analysis. Protein digests were analysed by a nano-liquid chromatography in a Proxeon Easy-nLC (Proxeon, Odense, Denmark) coupled to a MaXis quadrupole time-of-flight (Q-TOF) mass spectrometer (Bruker Daltonics, Bremen, Germany) according to Ošťádal et al. [38]. The samples of the peptide mixture were injected into an NS-AC-11-C18 Biosphere column (particle size 5 μm, length 150 mm, pore size: 12 nm, inner diameter 75 μm) with an NS-MP-10 Biosphere C18 precolumn (particle size 5 μm, length 20 mm, pore size: 12 nm, inner diameter 100 μm) (NanoSeparations, Netherlands).

Data were processed with the software ProteinScape v. 3.0.0.446 (Bruker Daltonics, Bremen, Germany). Proteins were identified by correlating tandem mass spectra to the extracted database for *Butyrivibrio fibrisolvens* from the NCBI database (downloaded on 26th February 2018; 33,312 proteins), using the MASCOT search engine v. 2.3.0 (http://www.matrixscience.com).

Trypsin was selected as the enzyme parameter. One missed cleavage was allowed, an initial peptide mass tolerance of ±10.0 ppm was used for MS and 0.05 Da for MS/MS analysis. Cysteines were assumed to be carbamidomethylated, proline and lysine to be hydroxylated, serine, threonine and tyrosine to be phosphorylated and methionine was allowed to be oxidized. All of these possible modifications were set to be variable. Monoisotopic peptide charge was set to 1+, 2+, and 3+. The Peptide Decoy option was selected during the data search process to remove false-positive results. The hits (MASCOT score ≥ 80, www.matrixscience.com) were accepted as significant. The metabolic pathways involving the observed enzymes were found using the website www.kegg.jp.

The isoelectric point of the proteins was estimated using the EMBOSS iep online tool (http://emboss.bioinformatics.nl/cgi-bin/emboss/iep). The molecular weight of the proteins was calculated using the online tool Protein Molecular Weight (http://www.bioinformatics.org/sms2/protein_mw.html). The carbohydrate metabolism proteins were identified using the Blast Koala online tool [28].

## Results

### Genome sequencing, theoretical proteome and enzyme assay

**Fig. 1:**
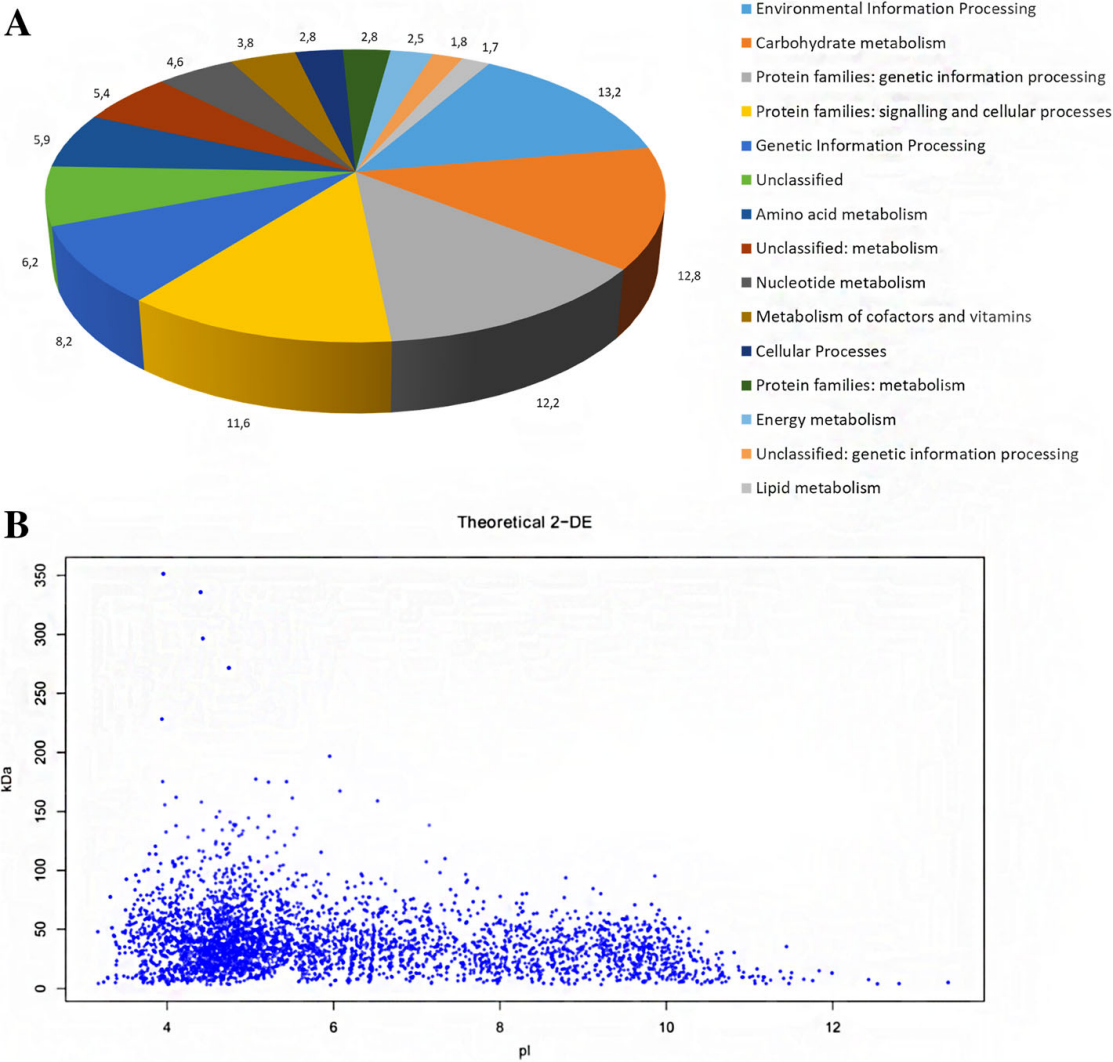
Protein function summary of all proteins identified in *B. fibrisolvens* 3071 (**a**) and theoretical 2DE map of total proteins (**b**). Blue spots represent all predicted proteins

The genome of *B. fibrisolvens* 3071 encodes 4079 coding sequences. According to the KEGG annotation, the major group of genes belonged to the Environmental Information Processing group (13.7%), followed by Carbohydrate metabolism genes (12.8%) and genes of Protein families for genetic information processing (12.5%), Protein families for signalling and cellular processes (11.1%) and Genetic Information Processing (11.3%). The Carbohydrate metabolism group includes 99 distinct Glycoside Hydrolase (GHs), 8 Carbohydrate Esterase (CEs) and 48 Glycoside Transferase (GTs) genes (Fig. 1a). Resulting annotated genome files (Additional file 1: Table S1) are in the Supplementary data.

The theoretical 2DE map constructed on the basis of the sequencing data indicates that the proteome of *B. fibrisolvens* 3071 consists of 4035 proteins. There were 2941 proteins (73%) possessing a theoretical p*I < 7*, the majority of them ranging between pI 4 and 7, other 1095 proteins (27%) had theoretical p*I* > 7 and only 172 proteins (4%) had pI > 10. Ninety-three percent of the predicted proteins (3754) had predicted polypeptide masses between 10 and 150 kDa (Fig. 1b).

**Fig. 2:**
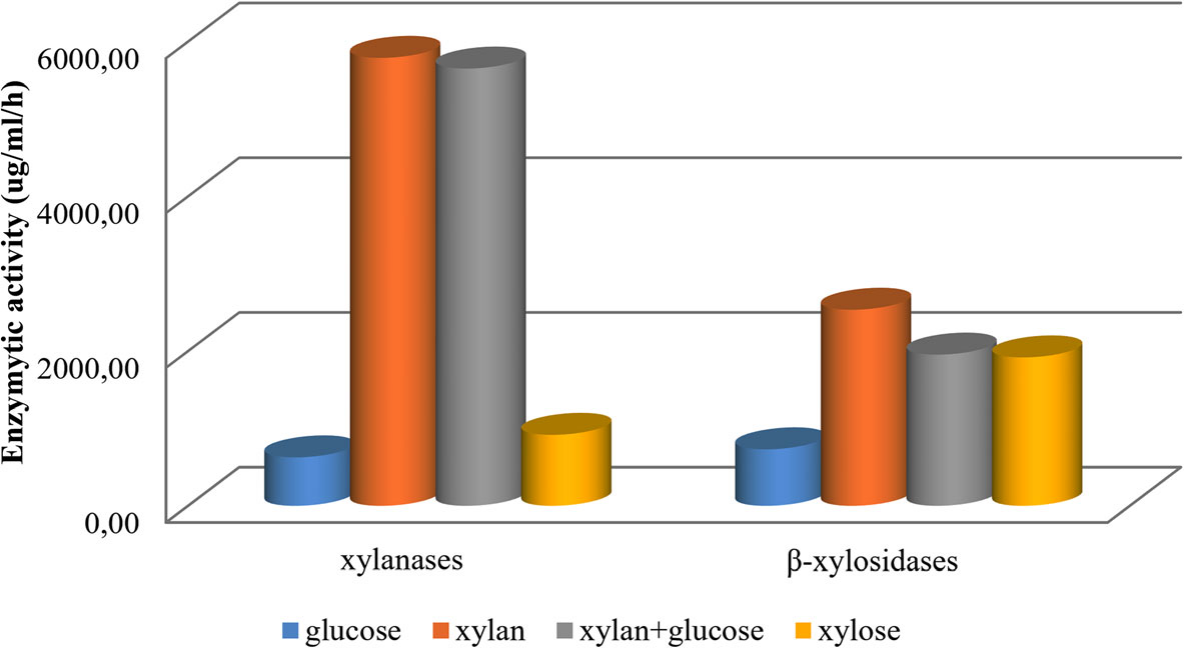
The comparison of xylanase and β-xylosidase activities of *B. fibrisolvens* 3071 grown on four different carbon sources: glucose (4 g/l), xylan (4 g/l), xylan (3 g/l) + glucose (1 g/l), and xylose (4 g/l)

Seven genes of endo-β-1,4-xylanase (EC 3.2.1.8) and 8 genes of xylan 1,4-β-xylosidase (EC 3.2.1.37) were detected in the genome. To verify the extracellular xylanolytic potential of strain 3071, the activities of xylanase, cleaving β-1,4- xylosidic linkages randomly within the xylan chain, and the activities of xylosidase, which removes successive xylose residues from the non-reducing termini, were investigated in the culture medium of *B. fibrisolvens* 3071 grown on four different substrates. Figure 2 shows that the production of both enzymes is dependent on the nature of the carbon source. Both enzymes were induced by the presence of xylan, especially xylanase, while xylosidase was also induced by monomeric xylose.

### SDS PAGE, 2-D electrophoresis and protein characterization

**Fig. 3:**
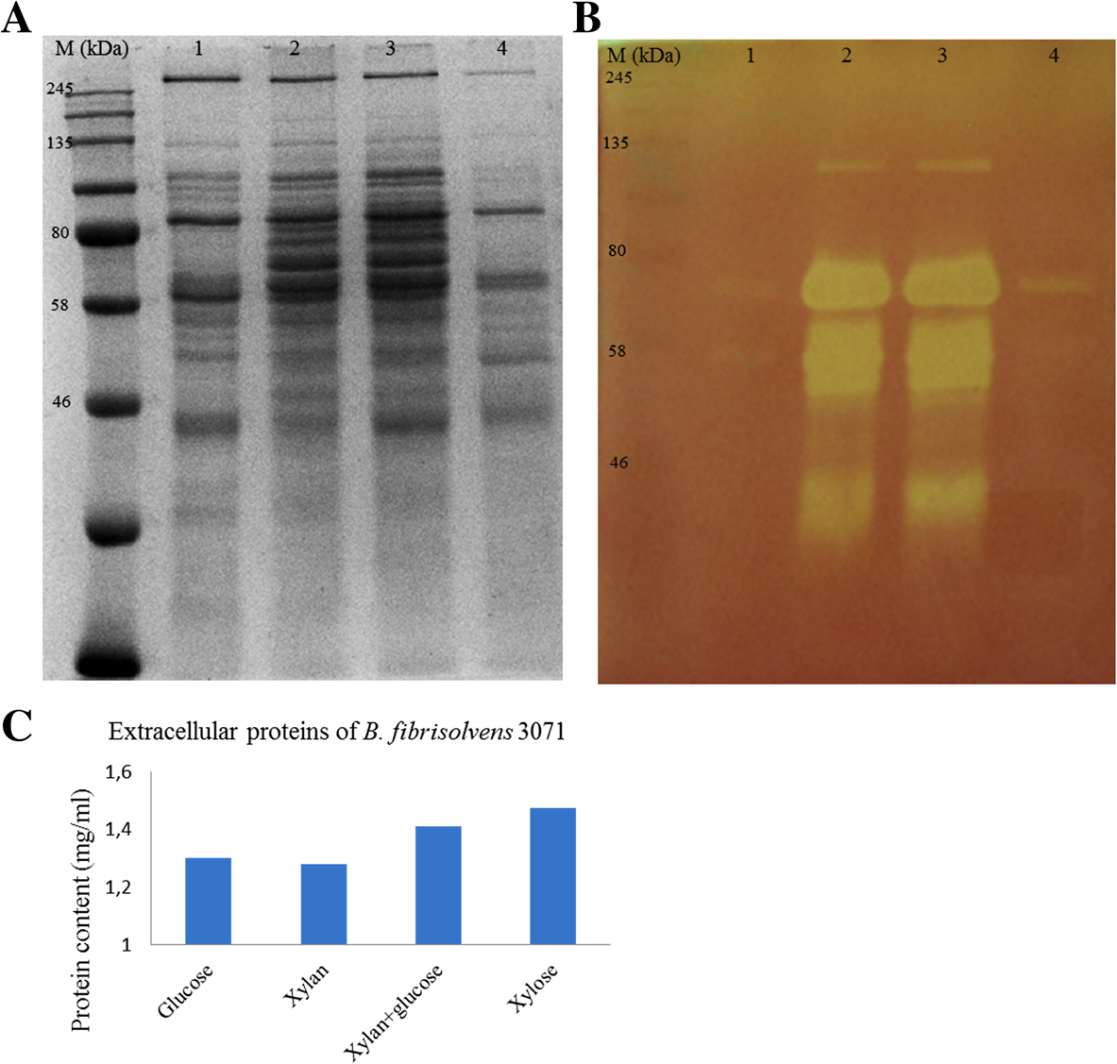
SDS PAGE (**a**) and zymogram (**b**) of extracellular proteins of *B. fibrisolvens* 3071. Strain was grown on the following carbon sources: 1 - glucose, 2 - xylan, 3– xylan and glucose, 4 – xylose. The MW protein standard was in the range 11–245 kDa. The protein concentrations in samples loaded onto both gels above is illustrated by blue columns (**c**)

SDS-PAGE analysis of the extracellular protein pattern of *B. fibrisolvens* 3071 grown on four different carbon sources revealed the major protein bands, with a higher intensity of estimated masses from 40 to 80 kDa when grown on xylan and a combination of xylan and glucose. Several protein bands of 58 to 80 kDa were visible for the strain cultivated on xylan and xylan+glucose, while these fragments were absent for cultivation on glucose and xylose. The zymogram shows differences in xylanase isoenzymes, in the range from 32 to 130 kDa, when xylan and xylan+glucose were used as the substrate. Only weak bands at 70 and 58 kDa were detected on the zymogram for enzymes from the strain grown on glucose and xylose (Fig. 3).

**Fig. 4:**
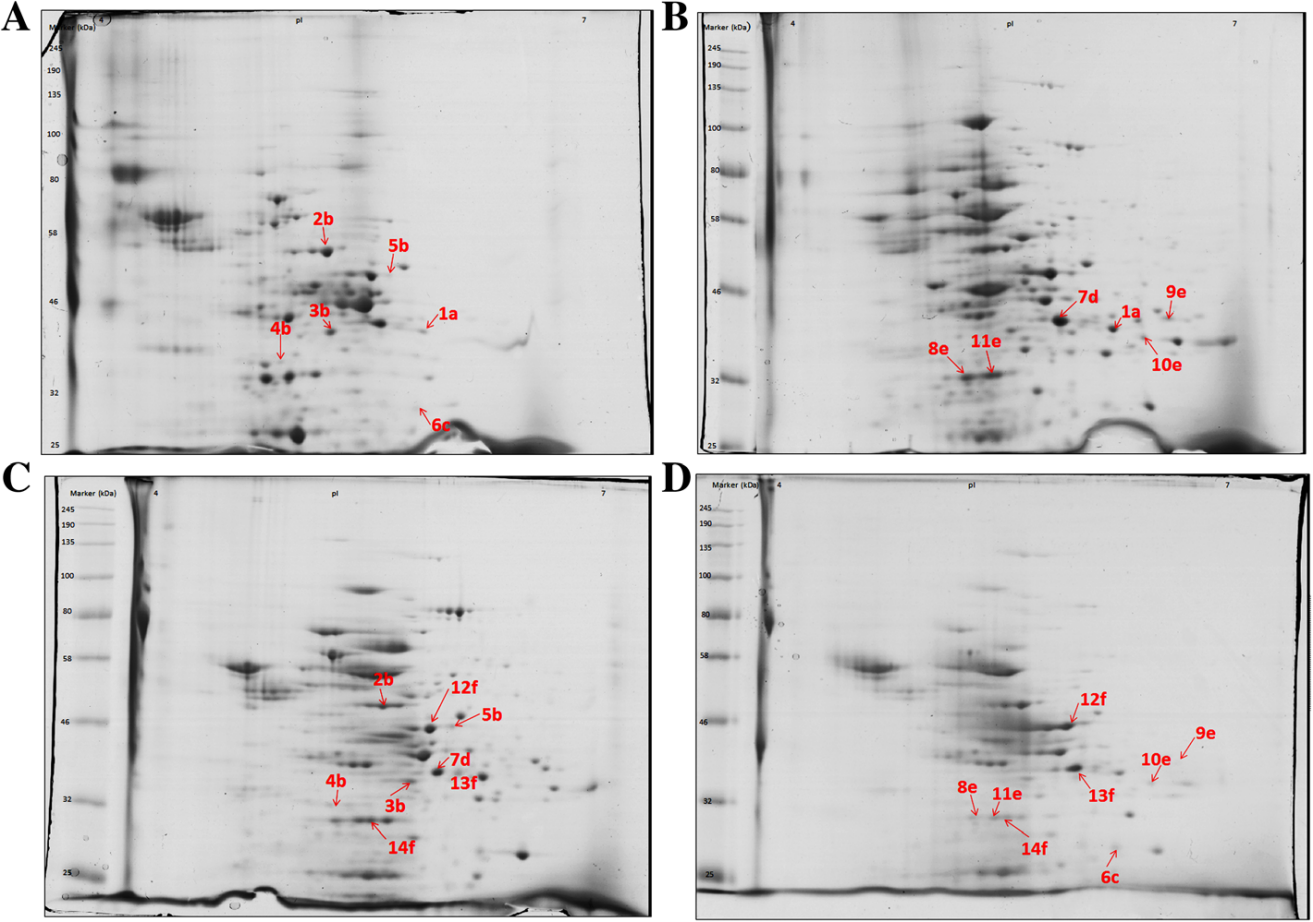
Two-dimensional gel electrophoresis of extracellular proteins of *B. fibrisolvens* 3071 grown on different substrates. The gels (**a**, **b**, **c**, **d**) show spots from cultivation on glucose, xylan, xylan + glucose, and xylose, respectively. The letters a, b, c, d, e, f indicate the identical spots, however with significantly different intensity, in the comparison of *B. fibrisolvens* 3071 grown on glucose/xylan, glucose/xylan + glucose, glucose/xylan + glucose, xylan/xylan+glucose, xylan/xylose, xylan+glucose/xylose, respectively. Annotations correspond to spot numbers in Table 1. The MW protein standard was in the range 11–245 kDa

Two-dimensional electrophoreses were carried out to identify the protein spot profiles of *B. fibrisolvens* 3071 grown on four different carbon sources. Substrates were compared to each other, amounting to six comparisons: (I) glucose/xylan, (II) glucose/xylan + glucose, (III) glucose/xylose, (IV) xylan/xylan + glucose, (V) xylan/xylose, (VI) xylan + glucose/xylose. Each comparison was performed in duplicate. From these eight 2-D electrophoresis gels, a total of 141 spots were excised and processed. Analysis of the nLC/MS protein peaks revealed that only 14 proteins (28 spots) fulfilled the MASCOT Score Criteria and were found as differentially expressed depending on the used carbon source, as shown in Fig. 4 and summarized in Table 1. These groups of proteins are involved in glycolysis, protein synthesis and butyrate synthesis. The identified proteins were functionally categorized as hydrolases (2), lyases (1), isomerases (1), transferases (3), oxidoreductases (2) and 3 proteins matched with Elongation factor Ts. The functions of these proteins and their involvement in the metabolic pathway are described in Table 2.

**Table 1:** List of *B. fibrisolvens* 3071 proteins identified and confirmed by nLC/MS as significantly different. The spots were excised from the 2-D electrophoresis gels comparing glucose/xylan (I), glucose/xylan + glucose (II), glucose/xylose (III), xylan/xylan + glucose (IV), xylan/xylose (V), xylan + glucose/xylose (VI)

Substrate comparison	Spot number	Accession number	Protein	Total number of peptides	Fold change	MW	pI
I	1a	SHI27642.1	diaminopimelate dehydrogenase	3	3.99 ↑g^1^	36.2	5.5
II	2b	SHH89614.1	glucose-6-phosphate isomerase	18	0.61 ↑x + g^3^	56.9	5.1
	3b	SHI23166.1	N-acetylmuramoyl-L-alanine amidase	6	0.44 ↑x + g^3^	38.9	5.2
	4b	SHH52657.1	1-phosphofructokinase	9	0.61 ↑x + g^3^	32.6	4.7
	5b	SER24636.1	phosphoglycerate kinase	6	2.02 ↑g^1^	43.8	5.2
III	6c	BAD51424.1	beta-hydroxybutyryl-CoA dehydrogenase	7	2.29 ↑g^1^	31.5	5.7
IV	7d	SHI29611.1	fructose-bisphosphate aldolase, class II	19	0.61 ↑x + g^3^	33.9	5.2
V	8e	SHI15246.1	elongation factor Ts	13	0.81 ↑xyl^4^	34.6	4.8
	9e	SHI83999.1	glyceraldehyde 3-phosphate dehydrogenase	8	0.88 ↑xyl^4^	36.9	5.8
	10e	SHH82532.1	3-deoxy-D-arabinoheptulosonate-7-phosphate synthase	2	0.69 ↑x^2^	38.5	6.0
	11e	SHI15246.1	elongation factor Ts	13	0.93 ↑x^2^	34.6	4.8
VI	12f	SER24636.1	phosphoglycerate kinase	2	2.22 ↑x + g^3^	46.8	5.2
	13f	SER75936.1	fructose-bisphosphate aldolase, class II	7	1.04 ↑x + g^3^	33.9	5.2
	14f	SHI15246.1	elongation factor Ts	2	0.62 ↑xyl^4^	34.6	4.8

Superscript 1, 2, 3, and 4 indicate glucose, xylan, xylan and glucose, and xylose, respectively

**Table 2:** Classification and metabolic functions of proteins detected in this study. Proteins in italic are involved in glycolysis

Name	Enzyme Nomenclature	Class	Metabolic pathway (KEGG)	Function	Reference
diaminopimelate dehydrogenase	3.5.1.28	hydrolases	lysine biosynthesis. Metabolic pathway, Biosynthesis of secondary metabolisms, Biosynthesis of amino acids	cleaves the amide bond between N-acetylmuramoyl and L-amino acids in bacterial cell walls	Gao et al., 2017 [39]
*glucose-6-phosphate isomerase*	5.3.1.9	isomerases	glycolysis/Gluconeogenesis, Pentose phosphate pathway, Starch and sucrose metabolisms, Amino sugar and nucleotide sugar metabolisms, Metabolic pathway, Biosynthesis of secondary metabolisms, Microbial metabolism in diverse environments, Biosynthesis antibiotics, Carbon metabolisms	catalyses the conversion of D-glucose 6-phosphate to D-fructose 6-phosphate	Verhees et al., 2001 [40]
N-acetylmuramoyl-L-alanine amidase	3.5.1.28	hydrolases	cationic antimicrobial peptide CAMP (resistance)	cleaves the amide bond between N-acetylmuramoyl and L-amino acids in bacterial cell walls (preferentially: D-lactyl-L-Ala)	Loessner et al., 1995 [41]
*1-phosphofructokinase*	2.7.2.56	transferases	glycolysis/Gluconeogenesis, Metabolic pathway Biosynthesis of secondary metabolites, Microbial metabolism in diverse environments, Biosynthesis of antibiotics, Carbon metabolism, Biosynthesis of amino acids	catalysed the transfer of a phosphoryl group from adenosinetriphosphate to fructuose-6-phosphate to obtain adenosinetriphosphate and fructose-1,6-bisphophate	Kimmel et al., 2000 [42]
*1-phosphoglycerate kinase*	2.7.1.56	transferases	glycolysis/Gluconeogenesis, Metabolic pathway Biosynthesis of secondary metabolites, Microbial metabolism in diverse environments, Biosynthesis of antibiotics, Carbon metabolism, Biosynthesis of amino acids	conversion of 1,3-bisphosphate glycerate to 3-phosphoglycerate	Yon et al., 1990 [43]
beta-hydroxybutyryl-CoA dehydrogenase	1.1.1.157	oxidoreductases	–	catalyses the third step in the β-oxidation cycle, production of butyrate	Taylor et al., 2010[44]
*fructose-bisphosphate aldolase, class II*	4.1.2.13	lyases	glycolysis/Glycogenesis, Pentose phosphate pathway, Fructose and mannose metabolism, Methane metabolism, Metabolic pathway, Biosynthesis of secondary metabolites, Microbial metabolism in diverse environments, Biosynthesis of antibiotics, Carbon metabolism, Biosynthesis of amino acids	catalyses the reversible aldol cleavage or condensation of fructose-1,6-bisphosphate into dihydroxyacetone-phosphate and glyceraldehyde 3-phosphate	Thomson et al., 1998 [45]
elongation factor Ts	–	–	–	catalyses the enzymatic binding of aminoacylt-RNA to ribosomes	Schwartzbach and Spermulli, 1989 [46]
*glyceraldehyde-3-phosphate dehydrogenase*	1.2.1.12	oxidoreductases	–	responsible for the interconversion of 1,3-diphosphoglycerate and glyceraldehyde-3-phosphate, a central step in glycolysis and gluconeogenesis. Forms exist which utilise NAD (EC:1.2.1.12), NADP (EC:1.2.1.13) or either (EC:1.2.1.59)	Fillinger et al., 2000 [47]
3-deoxy-D-arabinoheptulosonate-7-phosphate synthase	2.5.1.54	transferases	phenylalanine, tyrosine and tryptophan biosynthesis, Metabolic pathway, Biosynthesis of secondary metabolites, Biosynthesis of antibiotics, Biosynthesis of amino acids, Quorum sensing	catalyses the metabolic reactions called the shikimate pathway responsible for the biosynthesis of the amino acids phenylalanine, tyrosine, and tryptophan	Hermann, 2001 [48]

**Fig. 5:**
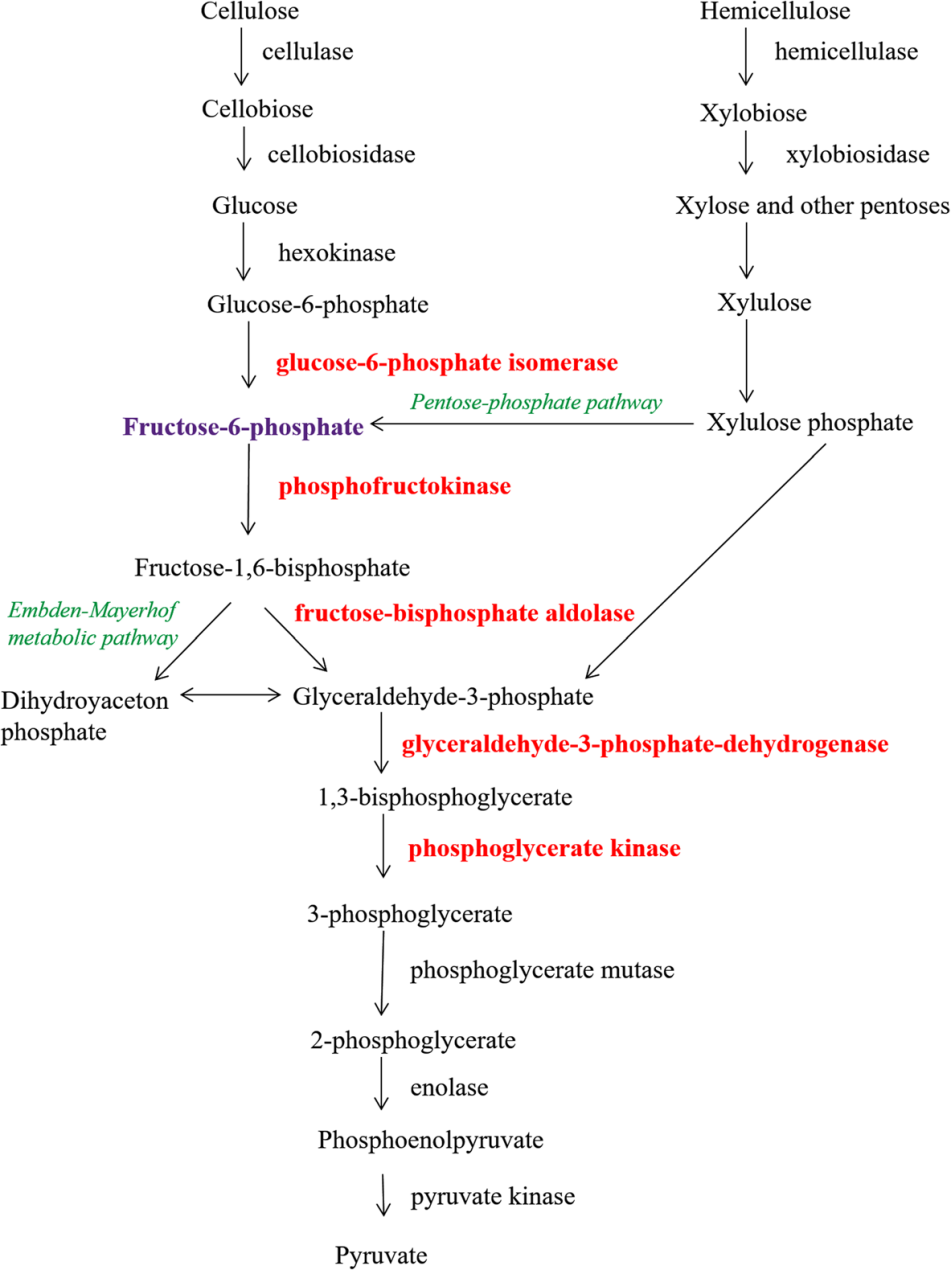
Scheme of metabolic pathway of decomposition of plant carbohydrates by *B. fibrisolvens* 3071. Red colour indicates enzymes detected in this study as spots with significantly different intensity depending on the carbon source

The enzymes involved in the process of degrading structural polysaccharides in the rumen were mapped using the KEGG database and the position and role of proteins of *B. fibrisolvens* 3071 in metabolic pathways were identified (Fig. 5).

## Discussion

*Butyrivibria* are considered to be an integral part of the bovine rumen bacteriome associated with the host genetic background, thus forming inheritable microbiota [2]. The biggest advantage of these bacteria is in the relatively broad spectrum of utilizable substrates and especially important is their xylanolytic activity [12]. The substrate flexibility of butyrivibria species was clearly documented by a study of the *B. proteoclasticus* glycobiome which covered a wide range of degrading and transporting proteins for different structural and storage polysaccharides [23], as well as a wide spectrum of oligosaccharides [24]. Due to the mobility of *Butyrivibria*, mediated by flagella, these strains also represent the most rapid colonizers of solid substrates in the rumen [4].

The sizes of genomes of *B. fibrisolvens* species sequenced to date range from 4462 up to 5121 kb. This corresponds well with our results, and shows that *B. fibrisolvens* as well as *B. proteoclasticus* [22, 49] have bigger genomes than other *Butyrivibria* and *Pseudobutyrivibria*. Their genomes have been determined in the range from 2100 to 4404 kb. In the *B. fibrisolvens* 3071 genome ten different endo-1,4-β-xylanase genes or precursors, and eight 1,4-β-xylosidase genes have been identified. The enzyme activity measurement and zymogram analysis confirmed that xylanase isoenzymes were in the extracellular fraction and were induced by xylan, even in the presence of a minor amount of glucose. These results are in agreement with the studies [50–53], however Sewell et al. [11] observed that xylosidase activity was mainly cell-associated and cellular xylanase activity rapidly declined in the presence of a small amount of glucose.

This study however mainly aimed to examine changes in the extracellular protein expression of *B. fibrisolvens* 3071 when this xylanolytic organism was grown on a variety of bioenergy-relevant substrates in order to identify the proteins responsible for substrate-specific breakdown and/or utilization. The substrates chosen ranged from simple (monomeric) to complex (polymeric), and varied in their general composition (C5 or C6 sugars). Proteomic analysis resulted in the highest number of spots when xylan was used as a substrate. Nearly all proteins were located in the central pI region. Comparison of proteome derived from simple sugars (glucose, xylose) exhibited a higher fold change in the proteins in the strain cultured on glucose, which indicates a tendency for a preferred sugar, which in the rumen is generally glucose, released by the hydrolysis of polysaccharides [18]. Xylose is not as efficiently utilized as glucose, which is caused by the different metabolic pathways of pentose and hexoses [54]. Pentose sugar represents about one-third of the total carbohydrate monomers in lignocellulosic biomass, which is a mixture of carbohydrate polymers (mainly cellulose and hemicellulose) and lignin [55]. The model of hemicellulose degradation by *Butyrivibria* was suggested by Palevich et al. [56] showing that the unsubstituted xylan chain is mainly degraded to xylose, which is transferred into the bacterial cell via xylose ABC transporters. Substituted xylo-oligosaccharides are transported by other ABC transporters and degraded by specific glycosidases in the cell cytoplasm.

In contrast to the results of Dunne et al. [24], who described debranching arabinofuranosidases, esterases, endo-β-1,4-xylanase and β-xylosidase enzymes in intracellular proteome *Butyrivibrio proteoclasticus* cultures, we did not identify the xylanolytic enzymes as significantly different with the carbon source used in this study. However, the design of our experiment was differed from study of Dunne et al. [24]. We did not study the whole proteome, but we aimed to compare the proteins which were differentially produced by *B. fibrisolvens* 3071 cultivated on different carbon sources.

In our work a significant regulation was observed in the extracellular fraction of groups of proteins involved in glycolysis and protein synthesis. The proteins affected in our analysis are the same as identified by Snelling and Wallace [21], who explored the ruminal digesta proteome of cattle, sheep and reindeer. These authors identified the glyceraldehyde-3-phosphate dehydrogenase, phosphoenolpyruvate carboxykinase, phosphoglycerate kinase and triosephosphate isomerase as a dominant enzymes of ruminal bacterial proteome. Two of these enzymes were also detected in our work as differentially expressed depending on carbon source. We also detected enzymes fructose-1,6-bisphosphate aldolase, glucose-6-phosphate isomerase involved in glycolysis. Snelling and Wallace [21] detected the beta-hydroxybutyryl-CoA dehydrogenase of *B. fibrisolvens* as the abundant prokaryotic protein in the rumen. These results support the high importance of *B. fibrisolvens* in the rumen ecosystem in spite of its low number [57]. The proteins involved in glycolysis, protein synthesis and carbohydrate metabolism were identified as the predominant pathways also by Hart et al. [58], who moreover also described the protein family containing the elongation factor Tu as being the most highly abundant in the bovine rumen ecosystem. Upregulated proteins of central carbon catabolic pathways have also been described by *Ruminiclostridium cellulolyticum* cultivated on different sources of fibre [59]. We find very interesting that proteomic studies performed on rumen fluid of different animals [21, 58] brought the same results as our work studying one pure bacterial strain. In our opinion this can indicate that the proteins of glycolysis, which provides sources energy to host animal, play a very crucial role and are essential for the metabolism of ruminants.

## Conclusion

This study was focused on the effect of substrates (glucose, xylan, xylan and glucose and xylose) on the expression of extracellular proteins. In *B. fibrisolvens* 3071 cultures, both extracellular β-endoxylanase activity and xylan β-xylosidase were induced by xylan. Xylan induced several hemicellulolytic isoenzymes in the interval from 32 to 130 kDa.

Proteomic analysis comparing complex and simple carbon sources revealed only limited numbers of significantly differently expressed proteins. These proteins are involved in glycolysis, protein synthesis and butyrate synthesis. Such a result was unexpected but not exceptional. Higher levels of the enzymes from the central carbon catabolic pathways were obtained in a bacterial monoculture of *Ruminiclostridium cellulolyticum* [59], as well as in the whole rumen microbial community [21, 58]. This is the first proteomic study of *B. fibrisolvens* 3071 indicating that this strain can play the important role for the central rumen catabolic metabolism.

## Additional files

Additional file 1: **Table S1.** Genome of *Butyrivibrio fibrisolvens* 3071 sequenced by PacBio annotated with Blast Koala (KEGG). (DOCX 15 kb)

Additional file 2: **Table S2.** Results of statistic evaluation ( Student’s test) and six pair-wise comparisons of 2D gels separating proteins of B. fibrisolvens 3071 grown on four different substrates (I – glucose, II - xylan, III xylan+glucose, 4 – xylose). **Figure S1.** 2D gel separation of proteins of *B.fibrisolvens* 3071 cultivated on glucose (I). **Figure S2.** 2D gel separation of proteins of *B.fibrisolvens* 3071 cultivated on xylan (II). **Figure S3.** 2D gel separation of proteins of *B.fibrisolvens* 3071 cultivated on glucose (I). **Figure S4.** 2D gel separation of proteins of *B.fibrisolvens* 3071 cultivation on xylan+glucose (III). **Figure S5.** 2D gel separation of proteins of *B.fibrisolvens* 3071 cultivated on glucose (I). **Figure S6.** 2D gel separation of proteins of *B.fibrisolvens* 3071 cultivated on xylose (IV). **Figure S7.** 2D gel separation of proteins of *B.fibrisolvens* 3071 cultivated on xylan (II). **Figure S8.** 2D gel separation of proteins of *B.fibrisolvens* 3071 on xylan+glucose (III). **Figure S9.** 2D gel separation of proteins of *B.fibrisolvens* 3071 cultivated on xylan (II). **Figure S10.** 2D gel separation of proteins of *B.fibrisolvens* 3071 cultivate on xylose (IV). **Figure S11.** 2D gel separation of proteins of *B.fibrisolvens* 3071 cultivated on xylan+glucose (III). **Figure S12.** 2D gel separation of proteins of *B.fibrisolvens* 3071 cultivated on xylose (IV). (DOCX 2222 kb)

## Abbreviations

2D: Two Dimensional
2DE: Two dimensional Electrophoresis
BPB: Bromophenol Blue
BSA: Bovine Serum Albumin
C5 sugar: Five Carbon Sugar
C6 sugar: Six Carbon Sugar
CCR: Carbon Catabolic Repression
CE: Carbohydrate Esterase
CHAPS: 3-[(3-cholamidopropyl) dimethylammonio]-1-propanesulfonate
CM: Carboxymethyl
DSMZ: Deutsche Sammlung von Mikroorganismen und Zellkuturen
DTT: Dithiothreitol
EC Number: Enzyme Commission Number
GH: Glycoside Hydrolase
GT: Glycoside Transferase
IA: Iodoacetamide
IEF: Isoelectric Focusing
IPG: Immobilized pH gradient
MS: Mass Spectrometry
MS/MS: Tandem Mass Spectrometry
MW: Molecular weight
nLC: Nano Liquid Chromatography
PAHBAH: 4-hydroxybenzoic acid hydrazide
PES: Polyethersulfone
*p*-NPX: p-Nitrophenyl-β-D-xylopyranoside
SDS-PAGE: Sodium Dodecyl Sulphate-Polyacrylamide Gel Electrophoresis
TGPA: Thermophilic Gram Positive Anaerobes
UK: United Kingdom
