# Supplementary material for: Comparison of enzymatic activities and proteomic profiles of Butyrivibrio fibrisolvens grown on different carbon sources
Source: Proteome Sci. 2019 Jun 1;17:2. doi: 10.1186/s12953-019-0150-3 (PMC6545216; doi:10.1186/s12953-019-0150-3)
Supplement: Supplementary file 1 — Table S1. Genome of Butyrivibrio fibrisolvens 3071 sequenced by PacBio annotated with Blast Koala (KEGG). (DOCX 15 kb) [file 12953_2019_150_MOESM1_ESM.docx]

**Table S1**: Genome of *Butyrivibrio fibrisolvens* 3071 sequenced by PacBio annotated with Blast Koala (KEGG).

|  | Sequence | Size (Mb) | GC % | Protein | rRNA | tRNA | Other RNA | Genes |
| --- | --- | --- | --- | --- | --- | --- | --- | --- |
| B.f. 3071 | [NZ_FQXK00000000.1](https://www.ncbi.nlm.nih.gov/nuccore/NZ_FQXK00000000.1) * | 4.84 | 39.70 | 4062 | 11 | 44 | 13 | 4130 |
| B.f. 3071 | *B. fibrisolvens*.gbk ** | 4.90 | 39.73 | 4081 | 10 | 46 | 13 | 4150 |

*The general available genome data of the *B. fibrisolvens* 3071 isolate were done by N. Varghese (DOE - Joint Genome Institute, CA) and are presented in GenBank accession FQXK01000003.1 (<https://www.ncbi.nlm.nih.gov/nuccore/NZ_FQXK00000000.1>).

**Genome of *B. fibrisolvens* 3071 processed in our lab (file enclosed).
