# Supplementary material for: Comparison of enzymatic activities and proteomic profiles of Butyrivibrio fibrisolvens grown on different carbon sources
Source: Proteome Sci. 2019 Jun 1;17:2. doi: 10.1186/s12953-019-0150-3 (PMC6545216; doi:10.1186/s12953-019-0150-3)
Supplement: Supplementary file 2 — Table S2. Results of statistic evaluation ( Student’s test) and six pair-wise comparisons of 2D gels separating proteins of B. fibrisolvens 3071 grown on four different substrates (I – glucose, II - xylan, III xylan+glucose, 4 – xylose). Figure S1. 2D gel separation of proteins of B.fibrisolvens 3071 cultivated on glucose (I). Figure S2. 2D gel separation of proteins of B.fibrisolvens 3071 cultivated on xylan (II). Figure S3. 2D gel separation of proteins of B.fibrisolvens 3071 cultivated on glucose (I). Figure S4. 2D gel separation of proteins of B.fibrisolvens 3071 cultivation on xylan+glucose (III). Figure S5. 2D gel separation of proteins of B.fibrisolvens 3071 cultivated on glucose (I). Figure S6. 2D gel separation of proteins of B.fibrisolvens 3071 cultivated on xylose (IV). Figure S7. 2D gel separation of proteins of B.fibrisolvens 3071 cultivated on xylan (II). Figure S8. 2D gel separation of proteins of B.fibrisolvens 3071 on xylan+glucose (III). Figure S9. 2D gel separation of proteins of B.fibrisolvens 3071 cultivated on xylan (II). Figure S10. 2D gel separation of proteins of B.fibrisolvens 3071 cultivate on xylose (IV). Figure S11. 2D gel separation of proteins of B.fibrisolvens 3071 cultivated on xylan+glucose (III). Figure S12. 2D gel separation of proteins of B.fibrisolvens 3071 cultivated on xylose (IV). (DOCX 2222 kb) [file 12953_2019_150_MOESM2_ESM.docx]

***Table S 2: Results of statistic evaluation ( Student´s test) and six pair-wise comparisons of 2D gels separating proteins of B. fibrisolvens 3071 grown on four different substrates (I – glucose, II - xylan, III xylan+glucose, 4 – xylose).***

***(Spot numbering in this table doesn´t correspond to the number of spot in Figure 4***

| **I vs. II** |  | **I vs. III** |  | **I vs. IV** |  | **II vs. III** |  | **II vs. IV** |  | **III vs. IV** |  |
| --- | --- | --- | --- | --- | --- | --- | --- | --- | --- | --- | --- |
| spot | T-test | spot | T-test | spot | T-test | spot | T-test | spot | T-test | spot | T-test |
| 11 | 0,067 | 11 | 0,455 | 1 | 0,238 | 1 | 0,059 | 53 (pouze v IV) | 0,286 | 10 | 0,173 |
| 1 | 0,501 | 2 | 0,336 | 10 | 0,057 | 10 | 0,262 | 1 | 0,707 | 1 | 0,530 |
| 22 | 0,097 | 1 | 0,422 | 22 | 0,263 | 2 | 0,269 | 10 | 0,531 | **11** | **0,045** |
| 21 | 0,131 | 10 | 0,070 | 21 | 0,286 | 11 | 0,913 | 2 | 0,484 | 2 | 0,277 |
| 20 | 0,890 | **20** | **0,014** | 20 | 0,975 | 20 | 0,514 | 20 | 0,487 | 58 | 0,407 |
| **19** | **0,020** | **19** | **0,003** | 14 | 0,723 | 14 | 0,611 | 54 | 0,073 | 14 | 0,341 |
| 14 | 0,576 | 14 | 0,514 | 11 | 0,388 | 19 | 0,592 | 11 | 0,182 | 4 | 0,325 |
| 11 | 0,722 | 4 | 0,075 | 19 | 0,212 | 4 | 0,233 | 14 | 0,194 | **3** | **0,004** |
| 4 | 0,535 | 3 | 0,421 | 18 | 0,346 | 46 | 0,808 | **19** | **0,029** | **19** | **0,006** |
| 3 | 0,969 | **5** | 0,030 | 17 | 0,891 | 47 | 0,129 | 4 | 0,268 | 5 | 0,052 |
| **5** | **0,042** | 18 | 0,147 | 16 | 0,049 | **3** | **0,041** | 3 | 0,324 | 15 | 0,060 |
| 6 | 0,181 | 17 | 0,209 | 6 | 0,078 | 5 | 0,588 | 12 | 0,132 | 24 | 0,404 |
| 7 | 0,678 | 16 | 0,102 | **38** | **0,047** | 18 | 0,408 | 13 | 0,449 | 57 | 0,202 |
| 8 | 0,677 | 15 | 0,070 | 39 | 0,300 | 17 | 0,408 | **7** | **0,020** | 18 | 0,626 |
| 9 | 0,745 | 25 | 0,493 | **37** | **0,042** | 16 | 0,128 | **8** | **0,033** | 17 | 0,279 |
| 12 | 0,377 | **12** | **0,019** | 40 | 0,443 | 48 | 0,960 | 9 | 0,845 | 16 | 0,218 |
| 13 | 0,392 | 13 | 0,293 | 41 | 0,545 | 49 | 0,217 | 37 | 0,088 | 53(only IV) | 0,174 |
| **15** | **0,027** | 7 | 0,298 | 7 | 0,303 | 50 | 0,246 | 55 | 0,051 | 6 | 0,310 |
| **16** | **0,041** | 8 | 0,302 | 8 | 0,088 | 15 | 0,092 | 39 | 0,395 | 38 | 0,548 |
| 17 | 0,655 | 9 | 0,439 | 9 | 0,274 | 13 | 0,958 | 38 | 0,528 | 39 | 0,501 |
| 18 | 0,182 | 17 | 0,243 | 23 | 0,185 | 7 | 0,861 | 6 | 0,601 | 37 | 0,535 |
| 23 | 0,543 | 6 | 0,228 | 43 | 0,268 | 8 | 0,142 | 5 | 0,141 | 7 | 0,162 |
| **24** | **0,039** | 26 | 0,171 | 42 | 0,554 | 9 | 0,579 | 15 | 0,052 | 8 | 0,066 |
|  |  | 27 | 0,122 | 13 | 0,461 | 37 | 0,118 | 18 | 0,183 | **9** | **0,033** |
|  |  | **28** | **0,028** | 12 | 0,114 | 51 | 0,324 | **17** | **0,034** | **20** | **0,010** |
|  |  | 29 | 0,114 | 4 | 0,395 | 52 | 0,554 | 16 | 0,228 | 13 | 0,358 |
|  |  | 30 | 0,774 | 3 | 0,131 | 6 | 0,449 | **24** | **0,041** | 59 | 0,092 |
|  |  | **31** | **0,049** | 30 | 0,346 |  |  | 57 | 0,842 | 60 | 0,403 |
|  |  | 32 | 0,084 | 15 | 0,434 |  |  |  |  | 31 | 0,946 |
|  |  | 33 | 0,117 | 44 | 0,112 |  |  |  |  |  |  |
|  |  | 34 | 0,129 | 45 | 0,452 |  |  |  |  |  |  |
|  |  | 35 | 0,156 | 46 | 0,346 |  |  |  |  |  |  |
|  |  | **36** | **0,040** |  |  |  |  |  |  |  |  |

**Comparison of 2D gels**

*Comparison I vs. II*


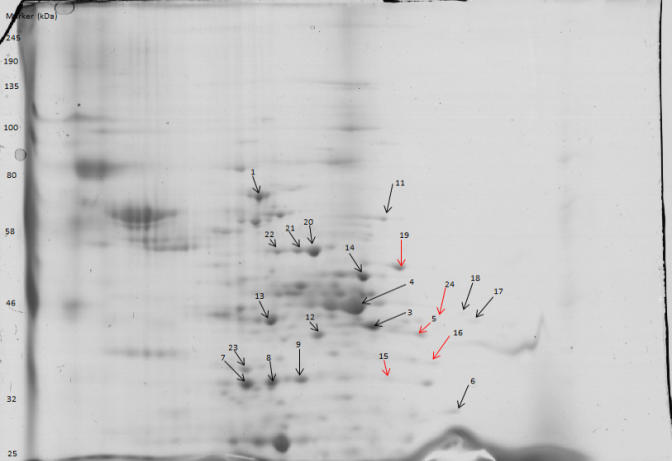


Figure 1: 2D gel separation of proteins of *B.fibrisolvens* 3071 cultivated on glucose (I)


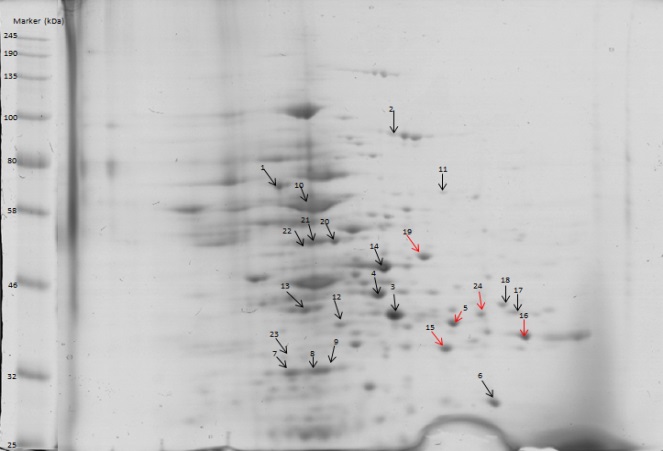


Figure 2: 2D gel separation of proteins of *B.fibrisolvens* 3071 cultivated on xylan (II)

*Comparison I vs. III*


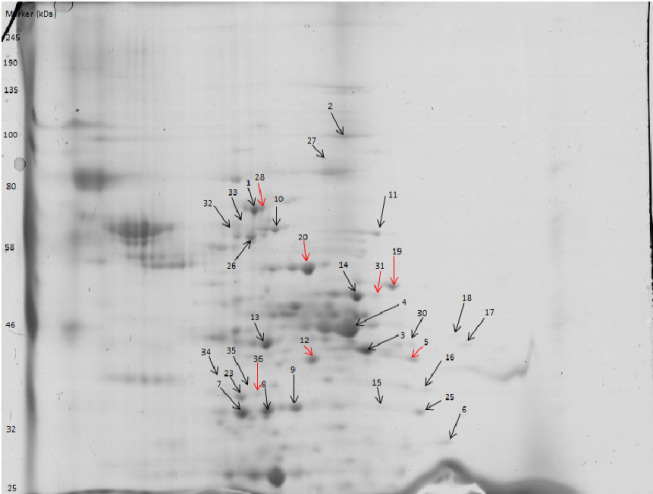


Figure 3: 2D gel separation of proteins of *B.fibrisolvens* 3071 cultivated on glucose (I)


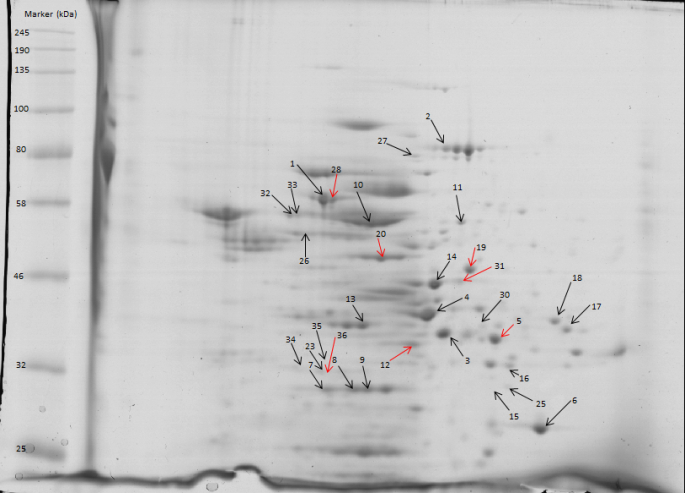


Figure 4: 2D gel separation of proteins of *B.fibrisolvens* 3071 cultivation on xylan+glucose (III)

*Comparison I vs. IV*


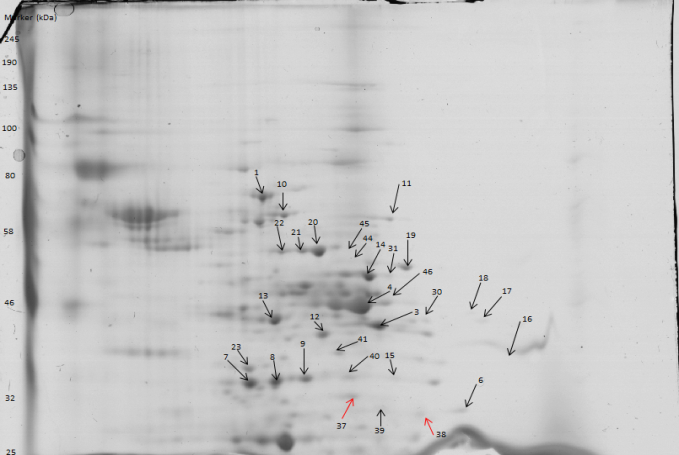


Figure 5: 2D gel separation of proteins of *B.fibrisolvens* 3071 cultivated on glucose (I)


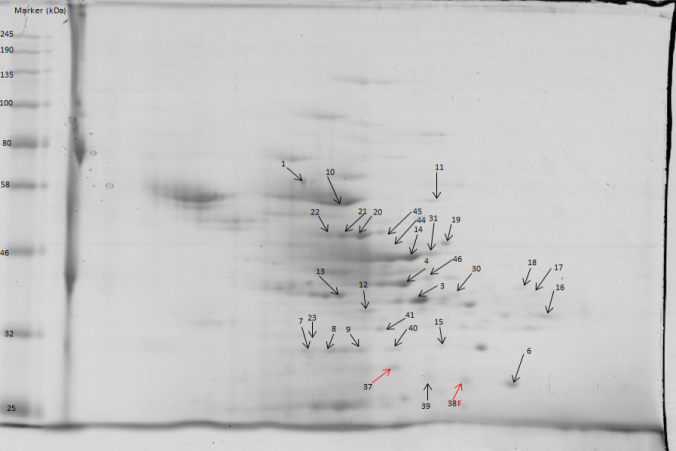


Figure 6: 2D gel separation of proteins of *B.fibrisolvens* 3071 cultivated on xylose (IV)

*Comparison II vs. III*


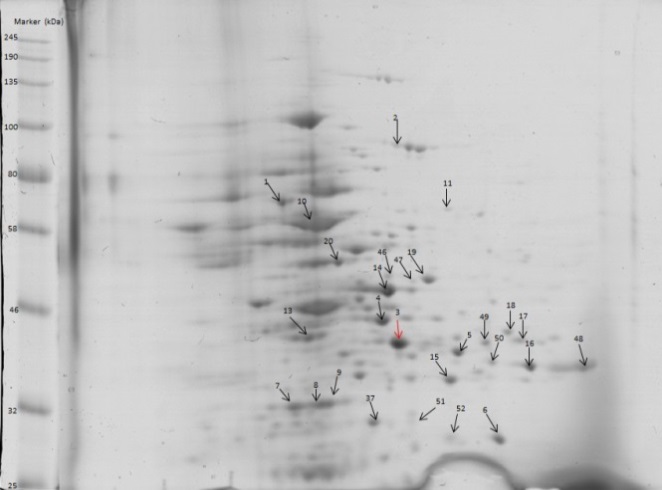


Figure 7: 2D gel separation of proteins of *B.fibrisolvens* 3071 cultivated on xylan (II)


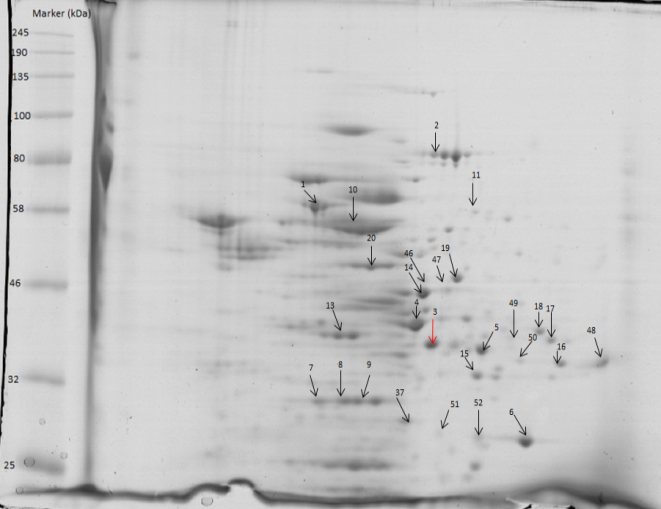


Figure 8: 2D gel separation of proteins of *B.fibrisolvens* 3071 on xylan+glucose (III)

*Comparison II vs. IV*


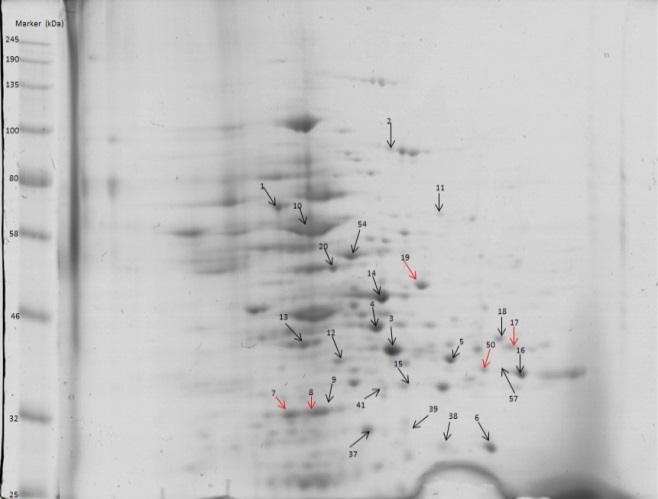


Figure 9: 2D gel separation of proteins of *B.fibrisolvens* 3071 cultivated on xylan (II)


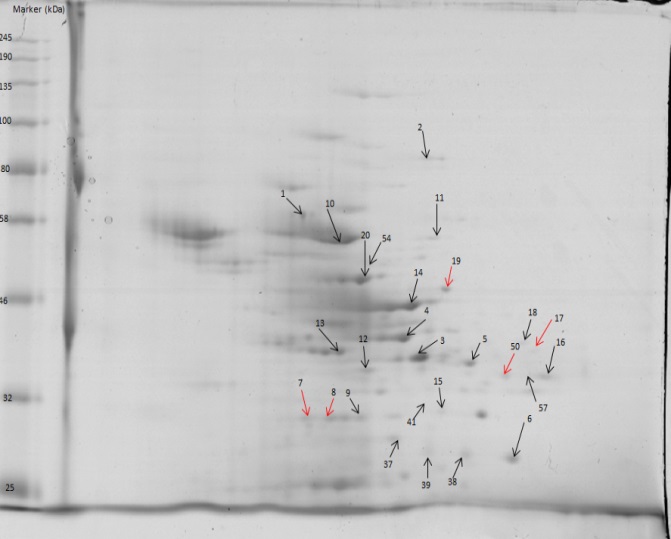


Figure 10: 2D gel separation of proteins of *B.fibrisolvens* 3071 cultivate on xylose (IV)

*Comparison III vs. IV*


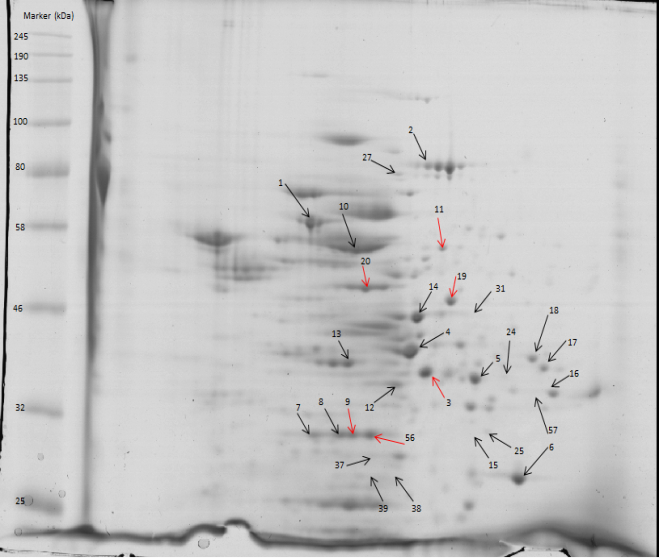


Figure 11: 2D gel separation of proteins of *B.fibrisolvens* 3071 cultivated on xylan+glucose (III)


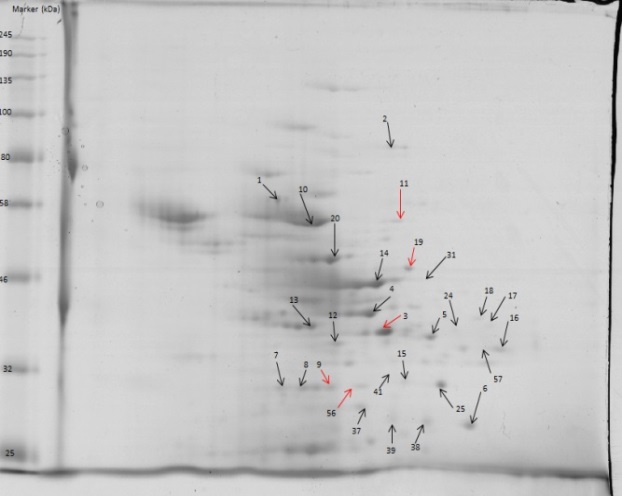


Figure 12: 2D gel separation of proteins of *B.fibrisolvens* 3071 cultivated on xylose (IV)
